# Bacterial Delivery of TALEN Proteins for Human Genome Editing

**DOI:** 10.1371/journal.pone.0091547

**Published:** 2014-03-11

**Authors:** Jingyue Jia, Yongxin Jin, Ting Bian, Donghai Wu, Lijun Yang, Naohiro Terada, Weihui Wu, Shouguang Jin

**Affiliations:** 1 Key Laboratory of Ministry of Education, Department of Microbiology, Nankai University, Tianjin, China; 2 The Key Laboratory of Regenerative Biology and The Guangdong Provincial Key Laboratory of Stem Cell and Regenerative Medicine, Guangzhou Institute of Biomedicine and Health, Chinese Academy of Sciences, Guangzhou, China; 3 Department of Pathology, University of Florida, Gainesville, Florida, United States of America; 4 Department of Molecular Genetics and Microbiology, University of Florida, Gainesville, Florida, United States of America; New England Biolabs, Inc., United States of America

## Abstract

Transcription Activator-Like Effector Nucleases (TALENs) are a novel class of sequence-specific nucleases that have recently gained prominence for its ease of production and high efficiency in genome editing. A TALEN pair recognizes specific DNA sequences and introduce double-strand break in the target site, triggering non-homologous end joining and homologous recombination. Current methods of TALEN delivery involves introduction of foreign genetic materials, such as plasmid DNA or mRNA, through transfection. Here, we show an alternative way of TALEN delivery, bacterial type III secretion system (T3SS) mediated direct injection of the TALEN proteins into human cells. Bacterially injected TALEN was shown to efficiently target host cell nucleus where it persists for almost 12 hours. Using a pair of TALENs targeting *venus* gene, such injected nuclear TALENs were shown functional in introducing DNA mutation in the target site. Interestingly, S-phase cells seem to show greater sensitivity to the TALEN mediated target gene modification. Accordingly, efficiency of such genome editing can easily be manipulated by the infection dose, number of repeated infections as well as enrichment of S phase cells. This work further extends the utility of T3SS in the delivery of functional proteins into mammalian cells to alter their characters for biomedical applications.

## Introduction

Transcription activator-like (TAL) effectors were first discovered in plant pathogen *Xanthomonas* sp. which directly injects the TAL effectors into plant cells through a type III secretion system (T3SS), where the TAL effectors specifically bind to and regulate plant genes to facilitate the bacterial colonization [1]. Each TAL effector contains a central region consisting of varying numbers of a repeating unit (average 34 amino acids), with each repeat specifically recognizing a particular DNA base. Accordingly, the DNA binding domain can be assembled using four types of repeats that recognize corresponding four nucleotides [2]–[4].

A novel class of sequence-specific nucleases have been generated by fusing the TAL effector to the catalytic domain of *Fok*I endonuclease, now called Transcription Activator-Like Effector Nucleases (TALENs) [5], [6]. TALENs are composed of a DNA binding domain that is capable of directing the *Fok*I nuclease to a specific target site. Two TALENs, recognizing left and right arms of the target site, respectively, are used to bring the two *Fok*I monomers close together for the formation of a functional dimer which generates DNA double-strand break (DSB) on the target site. The TALEN induced DSBs activate the DNA repair system within cells which stimulates “non-homologous end joining” in the absence of homologous DNA template. The error prone nature of this repair mechanism results in the introduction of nucleotide mismatches, insertions or deletions, often inactivating the target genes [7]. However, in the presence of a homologous template DNA, the DSBs triggers homologous recombination, introducing desired DNA sequence alterations.

The TALENs have rapidly gained prominence as a novel genome-editing tool, which were successfully applied to create site-specific gene modifications in the model organisms such as yeast, plants, zebra fish, rat, and even human pluripotent cells [8]–[12]. Delivery of the TALEN is currently based on introduction of genetic materials, such as DNA or mRNA, which is inefficient, especially for stem cells and iPS cells. Furthermore, introduction of DNA/RNA molecules poses the potential danger of integration-mediated mutagenesis as well as off target cleavage due to prolonged expression of the TALEN [13], [14]. All of these hinder clinical application of the TALEN technology. To overcome these shortcomings, a transient non-DNA or non-viral approach is highly desirable. Protein delivery serves as a safe alternative and there are a number of protein delivery technologies, such as fusions to cell penetrating peptide derived from Tat protein of retrovirus [15], [16], however, they are limited by the protein purification and low targeting efficiency.

*Pseudomonas aeruginosa* is a ubiquitous environmental bacterium which causes opportunistic human infections. T3SS of *P. aeruginosa* is a major virulence factor in establishing various host infections [17]. Upon activation, the T3SS translocates four protein effectors into the cytosols of host cells, including ExoS, ExoT, ExoY, and ExoU, causing cytotoxicity through different mechanisms [17]. Each type III injected protein is guided for delivery by the N-terminal secretion signal sequence [17]. We have previously shown that the first 54 amino acids of the *P. aeruginosa* exotoxin ExoS (ExoS54) is optimal in directing foreign proteins for injection through the bacterial T3SS [18], [19]. T3SS is highly efficient in rapidly injecting effector proteins into host cells and the efficiency of injection can easily reach 100% with MOI >20 in a short infection time (1–3 hours). An engineered protein delivery strain, deleted of all T3SS secreted effectors, does not show significant cytotoxicity and can easily be eliminated after the delivery by simple incubation with antibiotics [18], [19]. Since this naturally occurring protein injection machinery does not involve bacteria entering the host cells or DNA integration, *P. aeruginosa* is ideal for the delivery of exogenous proteins into mammalian cells for various purposes. Our previous studies have shown that the ExoS54 fused nuclear proteins can not only be successfully delivered into the mammalian cells but also efficiently targeted to nucleus where they exert their biological functions [18], [19].

In this study, we used type III secretion system of *P. aeruginosa* to deliver the ExoS54-TALEN fusion proteins into mammalian cells. Injected TALENs efficiently targeted to nucleus and accurately altered target gene sequence on the host chromosome, presumably through error prone repair of the double stranded breakages introduced by the TALENs. In this new delivery method, TALEN proteins are directly injected into the host cells, avoiding the introduction of foreign genetic materials (DNA/RNA). Also, due to the short half-life of the injected TALEN proteins, off-target effect should be minimized. These studies serve as a foundation for bacterial delivery of TALENs to efficiently and safely edit genome without jeopardizing genome integrity, fulfilling the basic safety requirement for medical application of the engineered cells.

## Results

### Bacterial T3SS Mediated Secretion of TALEN Proteins

**Figure 1:**
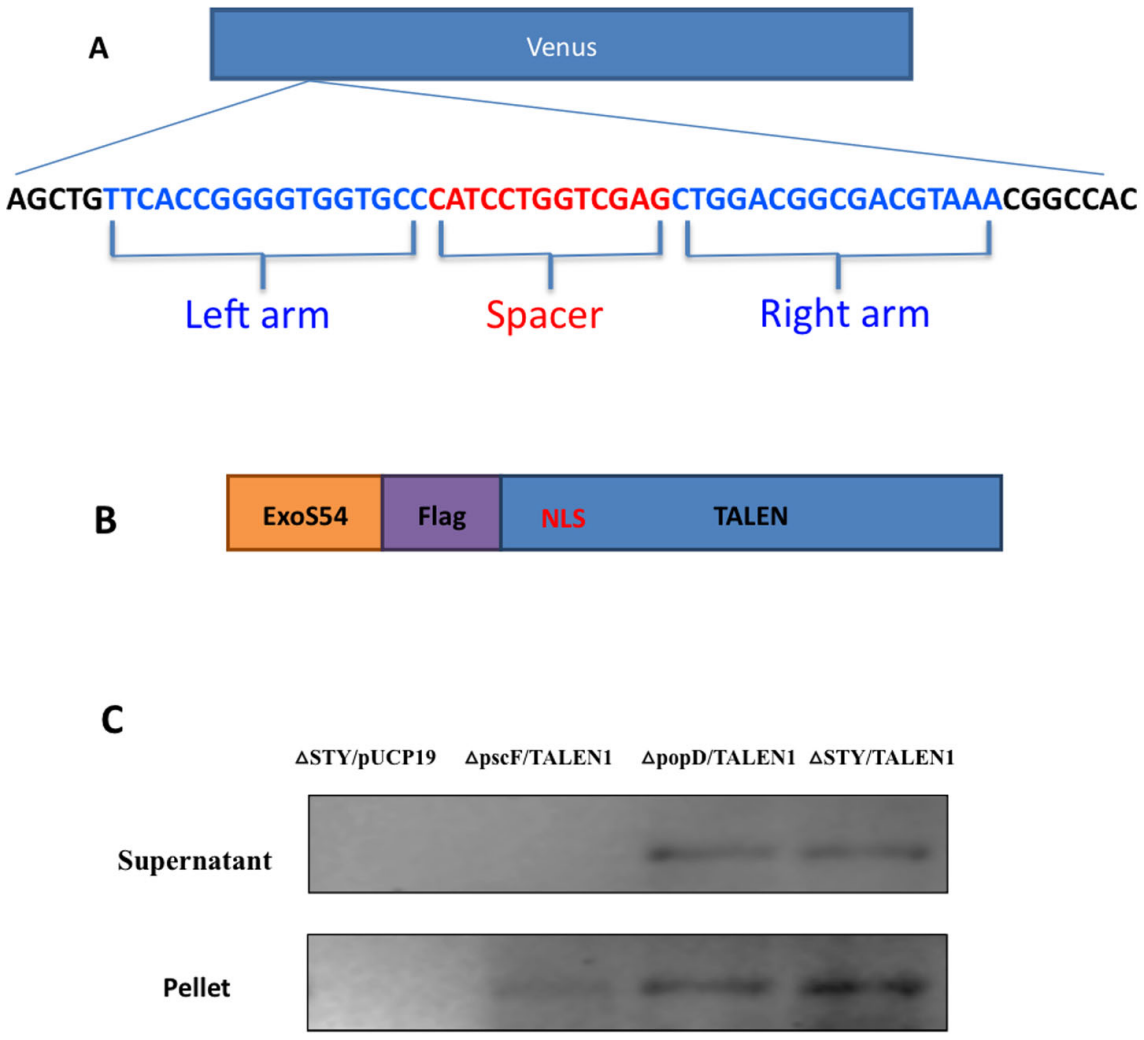
Bacterial secretion of TALEN fusion protein. (**A**) Diagram of TALEN binding sites on *venus* gene. (**B**) Diagram of ExoS54-Flag-TALEN fusion protein; NLS, nuclear localization sequence. (**C**) Secretion profiles of laboratory strains of PAK-JΔ*STY* containing pUCP19 vector or PAK-JΔ*pscF*, PAK-JΔ*popD* and PAK-JΔ*STY* harboring pExoS54-F-TALEN1. Strains were grown under type III secretion inducing condition and culture supernatants and pellets were subjected to SDS PAGE followed by Western blot using anti-Flag antibody.

A pair of TALEN constructs, targeting the gene encoding Venus fluorescent protein, have been generated using a Golden Gate cloning kit generated by Voytas laboratory [8]. The final TALEN1 and TALEN2 constructs, recognizing 17 bp of left and right arms (with 13 bp space) of the *venus* gene, respectively (Fig. 1A), were each cloned into a eukaryotic expression vector pTAL3 for delivery by plasmid transfection. To deliver TALEN proteins using bacterial T3SS, the two TALENs were cloned into pExoS54F-TAL where a bacterial promoter with N-terminal 54 amino acids of ExoS were fused to the TALEN with a FLAG-tag in the fusion junction (Fig. 1B). The amino-terminal 54 amino acids of *P. aeruginosa* exotoxin ExoS have previously been shown to be optimal for delivery of exogenous proteins into mammalian cells through the T3SS of *P. aeruginosa* [18], [19].

Plasmids pExoS54F-TALEN1 and pExoS54F-TALEN2, expressing the TALEN fusion proteins, were each electroporated into three different *P. aeruginosa* strains. Strain PAK-JΔ*STY* maintains high type III secretion capacity and diminished toxicity due to the deletion of endogenous exotoxins *exoS*, *exoT*, *exoY* [18], strain PAK-JΔ*pscF* is deleted of the type III needle structural gene, thus defective of the type III secretion [20], while strain PAK-JΔ*popD* is deleted of a gene encoding a protein required for the formation of translocon pore on the host membrane, thus unable to inject effectors into the host cells but still capable of protein secretion into culture medium [21]. The resulting transformants were cultured in L-broth in the presence of 5 mM EGTA for 3 hours to induce the type III secretion. The supernatants and cell pellets were separated by centrifugation and then subjected to Western blot analysis using anti-Flag antibody. The TALEN fusion proteins with expected sizes (100 KD) were detectable in the cell lysates of all strains (Fig. 1C), but the fusion proteins were only detectable in the culture supernatants of PAK-JΔ*STY* and PAK-JΔ*popD*, indicating that the fusion constructs were capable of directing the production of expected fusion proteins in the bacterial cells, but a functional T3SS is required for their secretion into the culture supernatants. The size of TALEN protein was more than 100 KD, which is the largest exogenous protein ever shown successfully secreted by the T3SS of *P. aeruginosa* thus far.

### Bacterial T3SS Mediated Injection of TALEN Proteins into HeLa Cells

**Figure 2:**
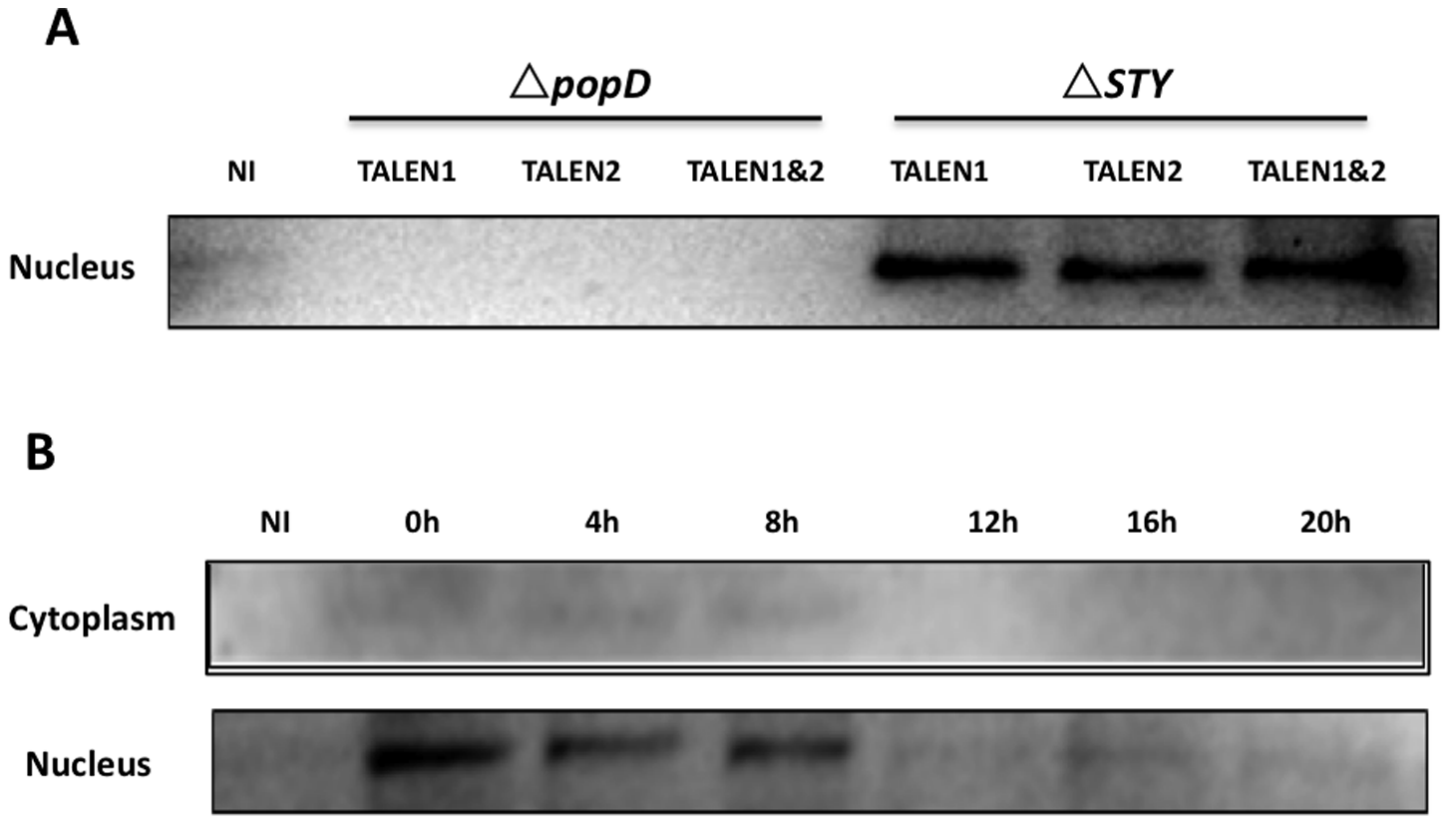
Intracellular localization and stability of the T3SS injected TALENs. (**A**) HeLa-Venus cells were infected with the indicated strains at an MOI of 100 for 3 hours. Nuclear proteins were extracted and subjected to SDS PAGE and Western blot by anti-Flag antibody; (**B**) HeLa-Venus cells were infected with PAK-JΔ*STY*/pExoS54-F-TALEN1 and PAK-JΔ*STY*/pExoS54-F-TALEN2 at MOI of 100 each for 3 hours. At the indicated time after the termination of infection, cytoplasmic and nuclear protein extracts were prepared and subjected to SDS PAGE and Western blot assay using anti-Flag Ab. NI, no infection control.

To assess the capacity of *P. aeruginosa* T3SS to inject TALEN proteins into host cells, a human cervical epidermal cell line (HeLa) was infected with PAK-JΔ*STY* harboring the ExoS54F-TALEN fusion constructs at a Multiplicity of Infection (MOI) of 100 for 3 hours, and extracts of nuclear proteins were prepared for Western blot analysis by anti-Flag antibody. To verify that the ExoS54F-TALEN injection occurs in a T3SS dependent manner, the T3SS defective strain PAK-JΔ*popD* was also used as a negative control. The MOI and infection time were chosen based on the experimental data in our previous studies [18], [19]. Cells were infected by each TALEN delivery strain at MOI of 100, thus the cells infected by both TALEN delivery strains had an overall MOI of 200. As the injection assay results shown in Fig. 2A, the TALEN fusion proteins were efficiently injected into the host cells by the *P. aeruginosa* in a T3SS dependent manner and the injected TALENs correctly localized to the nucleus.

To address the intracellular stability of the injected TALENs, cells were collected at various time points following injection at MOI of 100 for 3 hours. Cell lysates were fractionated into cytoplasmic and nuclear proteins, and subjected to Western blot using anti-Flag antibody. Quantitative analysis of the protein bands shown in Fig. 2B by Image-J program, revealed that more than 90% of the injected TALEN fusion proteins were quickly and efficiently localized to the nucleus after the initial 3 hour infection (time 0), followed by a gradual degradation in a time-dependent manner till sometime before 12 hours post infection. As the cell cycle of HeLa cells is about 24 h, the injected TALEN fusion proteins persist in the HeLa cell nucleus for almost half of the cell cycle.

The TALEN was based on the TAL protein of *Xanthomonas spp.* where HpaB chaperone facilitates the TAL injection into plant cells through the T3SS of *Xanthomonas spp* [22]. To test whether HpaB protein can facilitate the secretion of TALEN in *P. aeruginosa*, we constructed a plasmid expressing the *hpaB* gene and introduced it into the TALEN delivery strain of PAK-JΔ*STY*. Unfortunately, no obvious difference was observed in the efficiency of TALEN secretion or injection in the presence or absence of the HpaB expressing plasmid (data not shown). It is possible that either the HpaB is unable to interact with the T3SS machinery of *P. aeruginosa* or additional factors from *Xanthomonas spp.* are needed to fulfil the chaperone function of the HpaB protein.

### TALEN Delivery by Plasmid Transfection

**Figure 3:**
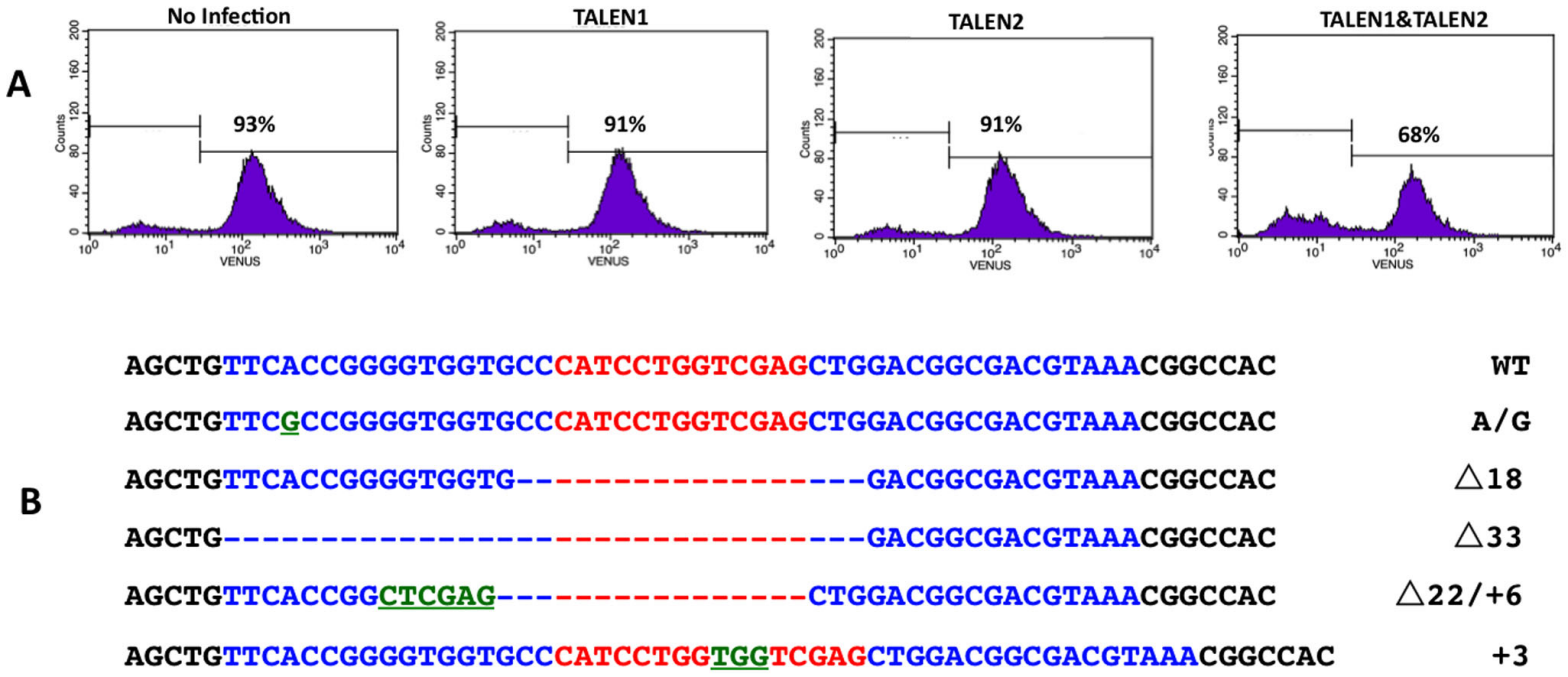
Plasmid transfection mediated delivery of TALENs into HeLa-Venus cells. (**A**) Fluorescent intensity of HeLa-Venus cells transfected by the indicated TALEN encoding plasmid constructs. Cells were analyzed by flow cytometry 3 days after the transfection. (**B**) Sequence changes in TALEN target region among the Venus negative cells.

To assess the functionality of the TALEN constructs, a stable Venus expressing HeLa cell line was generated by transfecting *venus* expressing plasmid (pBCAG-Venus) followed by puromycin selection and single cell cloning. A stable HeLa-Venus cell line was then transfected with a eukaryotic expression vector (pTAL3) encoding *venus* targeting TALEN. As two TALENs were designed to target left and right arms of the *venus* DNA sequence, correct binding of the two TALENs to their target sites will allow *Fok*I dimer formation which generates double-strand cleavage in the space between the two binding sites (Fig. 1A). The cells were transfected by single or double plasmids using liposome and then cultured under regular growth medium for 3 days. Judging from parallel transfection of a RFP expressing plasmid, the transfection efficiency was about 60%. Fluorescence of the cells was observed under microscope and clusters of cells diminished of the fluorescence were significantly more in cells transfected by both plasmids compared to those transfected with single TALEN construct. The fluorescent cell populations were further assessed by flow cytometry. Compared to the non-transfected cells or the cells transfected by single TALEN plasmid (targeting either right or left arm), HeLa-Venus cells co-transfected with both plasmids had approximately 20% cells lost of their fluorescence (Fig. 3A). To address if the loss of fluorescence was due to mutation in the TALEN target site, the non-fluorescent cells were sorted by FACS and total genomic DNA was purified. A 350 bp target region of the *venus* gene was PCR amplified and cloned into a TA cloning vector pGEM-T Easy. Ten clones were randomly chosen for sequencing. As the sequence result shown in Fig. 3B, five different mutations were identified around the target site of the TALENs, indicating that the two TALENs correctly recognized their target sites and introduced double stranded breaks, triggering error-prone repair that resulted in the indicated mutations. The remaining five had wild type *venus* sequence, likely derived from the spontaneous non-fluorescent cells due to epigenetic silencing of the exogenous *venus* gene.

### Functional Analysis of the Bacterially Injected TALEN Proteins

**Figure 4:**
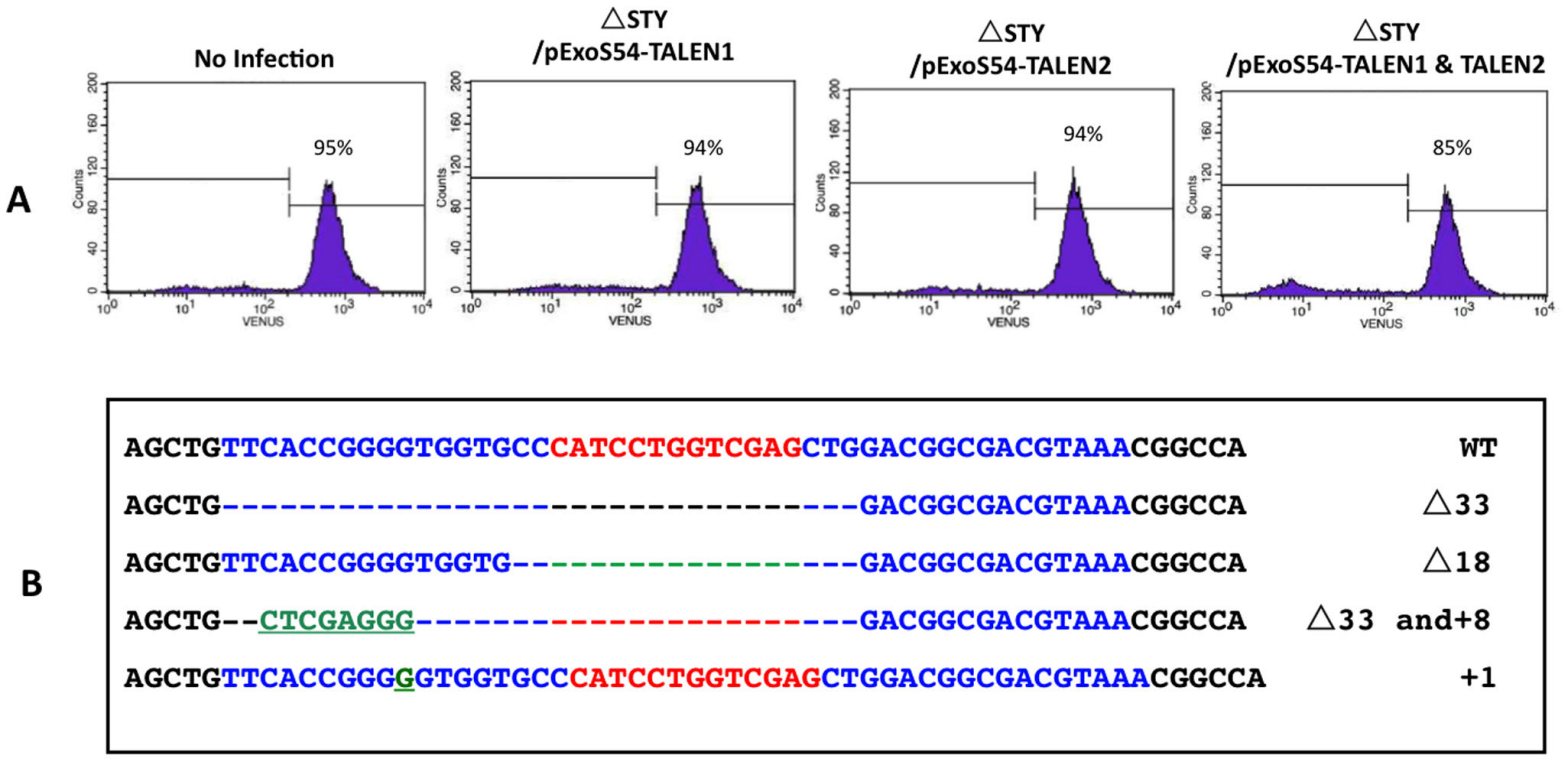
Loss of fluorescence in HeLa-Venus cells following bacterial delivery of TALEN proteins. (**A**) FACS analysis of fluorescence cell population three days after the delivery of indicated TALENs by *P. aeruginosa*; (**B**) Sequence changes in the TALEN targeting region among Venus negative cells.

To determine the functions of TALEN proteins delivered by the bacterial T3SS, the HeLa-Venus cells were infected by PAK-JΔ*STY* harboring each of the two pExoS54F-TALEN at a MOI of 100 for 3 hours and washed three times with PBS to remove floating bacterial cells. The HeLa-Venus cells were then grown in DMEM+10% FBS medium supplemented with 25 µg/ml ciprofloxacin to clear the residual bacterial cells. After 3 days of culturing, the cells were observed under fluorescent microscope and the fluorescent cell population was further monitored by flow cytometry. Compared to the uninfected control, we observed that significant number of cells lost of their fluorescence upon simultaneous delivery of both TALENs, with approximately 10% cells became non-fluorescent as assessed by FACS analysis (Fig. 4A). The fluorescence negative cells were further collected by flow cytometry, from both uninfected and those infected by the TALEN pair. Total genomic DNAs were extracted, PCR amplified the *venus* target sequences and cloned into the pGEM-T Easy vector. In the uninfected control cells, all 10 randomly chosen clones had wild type sequence. However, sequence analysis of 10 randomly chosen clones derived from the TALEN treated cells revealed four different mutation types on the target site (Fig. 4B), with the remaining six showing wild type sequences. Based on the precise locations of the *venus* gene mutations, we conclude that the TALENs delivered by T3SS of *P. aeruginosa* exerted their biological functions properly inside the nucleus of target cells.

### Repeated Delivery of TALEN Further Increased Percentage of Venus Negative Cells

**Figure 5:**
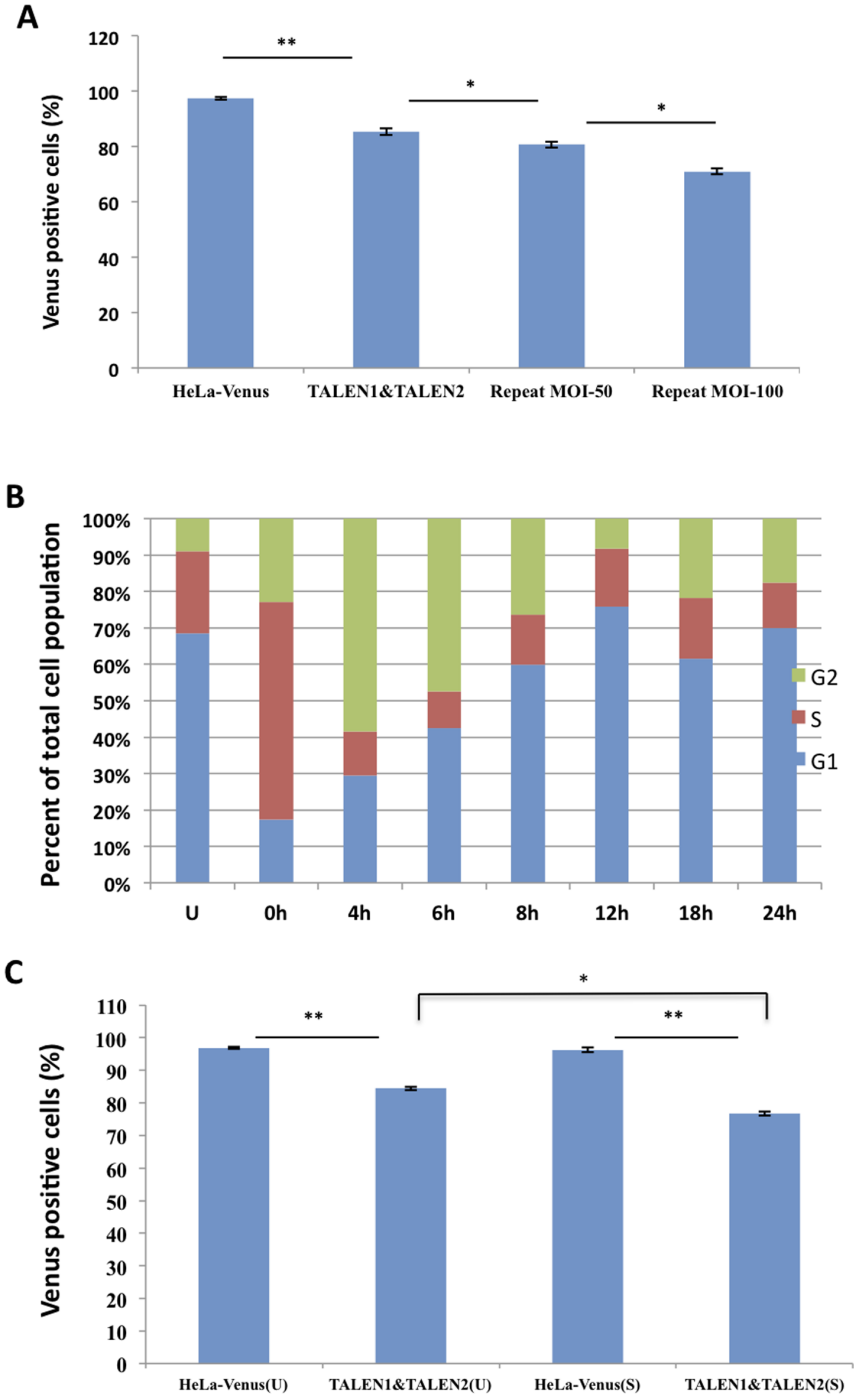
Improvement of TALEN targeting efficiency. (**A**) Multiple rounds of TALEN injection. HeLa-Venus cells were infected with PAK-JΔ*STY*/pExoS54-F-TALEN1 and PAK-JΔ*STY*/pExoS54-F-TALEN2 at MOI of 100 each for 3 hours. Bacteria were cleared and cells were cultured for 24 hours, then repeated injection at MOI of 50 or 100. The FACS data of infected cells were compared by paired t-test and ANOVA. *P<0.05; **P<0.001. Error bars indicate standard deviations of triplicate assays. (**B**) HeLa-Venus cells were either left unsynchronized (U) or synchronized (S) through double thymidine blocking and released to determine the duration of the cell cycle by FACS analysis. (**C**) HeLa-Venus cells were synchronized to S phase (S) or unsynchronized (U) and infected by PAK-JΔ*STY*/pExoS54-F-TALEN1 and PAK-JΔ*STY*/pExoS54-F-TALEN2 at MOI of 100 each for 3 hours. The FACS data of infected cell populations were compared by paired or two-sample t-test. *P<0.05; **P<0.001. Error bars indicated the standard deviations of triplicate assays.

Given that the target gene can be knocked out at an efficiency of 10% by single injection of the TALEN pair, multiple rounds of protein injection may further improve the efficiency of target gene knockout. To test this, HeLa-Venus cells were infected at an MOI of 100 for 3 hours, then the infections were cleared and cells were cultured in regular growth medium supplemented with 25 µg/ml ciprofloxacin for 24 hours. Second round of injection was conducted at MOI of either 50 or 100 for 3 hours, followed by three days of growth in the presence of ciprofloxacin before subjecting to analysis by flow cytometry. When the second round of infection was carried at MOI of 100, greater than 20% of the resulting cells lost fluorescence, more than those infected at MOI of 50 which resulted in 14% Venus negative cells (Fig. 5A). The above results indicated that multiple rounds of protein injection can indeed enhance the efficiency of *venus* target knockout and the increase is proportional to the infection dose (MOI).

### Influence of Cell Cycle on TALEN Mediated Gene Targeting

It is conceivable that TALEN-mediated gene knockout can only occur when the target sites are freely accessible, most likely during the DNA replication or S phase of the cell cycle. If that is true, the efficiency of gene knockout can be enhanced if the S-phase cells are enriched. To test for this, HeLa-Venus cells were synchronized by a double thymidine blocking method and released at various time points [18] (Fig. 5B). The synchronization experiment indicated that the duration of cell cycle under our growth condition is around 24 hours. Following synchronization, the S phase cells can be enriched more than 50% of the cell population, compared to 20% S-phase cells in unsynchronized cells. HeLa-Venus cells synchronized to S-phase or unsynchronized were infected with the TALEN pair delivery strains at MOI of 100 for 3 hours, followed by three days of culture, and then measured the percentages of Venus negative cells. As the results shown in Fig. 5C, compared to the uninfected controls, the proportion of Venus negative cells in synchronized cells reached approximately 20% while that of unsynchronized cells remained about 10%, suggesting that S-phase cells are more susceptible to TALEN mediated target cleavage, presumably due to an easier access of TALENs to their target sites.

## Discussion

TALEN technology is a powerful tool in creating knockouts in various eukaryotic model organisms, however, the TALENs are currently delivered into the host cells in the form of plasmid DNA or RNA, posing the potential danger of exogenous genetic materials integrating into the genome of target cells. A much safer alternative is to directly deliver TALENs in the form of proteins into the host cells. *P. aeruginosa* naturally injects several toxic proteins into eukaryotic cells via its type III secretion system (T3SS), and we have successfully utilized this system to deliver exogenous proteins into the host cells to exert their biological function, such as Cre recombinase to trigger LoxP site mediated recombination [18] and MyoD mediated trans-differentiation of fibroblasts into myocytes [19]. In both cases, the bacterially injected proteins targeted to the nucleus via nuclear localization sequence and exerted their biological functions through interaction with the host chromosomal DNA targets. In the current study, we fused the N-terminal 54 amine acids of ExoS with TALENs designed to target *venus* gene and observed that bacterially injected ExoS54F-TALEN fusion proteins not only correctly targeted to the nucleus but also efficiently exerted their biological functions, *i.e.* successfully knocked out the target *venus* gene on the mammalian chromosome, presumably through double-stranded breakage (DSB) and error-prone DNA repair. Sequence analysis of the TALEN target site among the Venus negative cells further confirmed DNA mutation around the predicted DSB site (Fig. 4B). The bacterially delivered TALEN achieves the goal of editing mammalian genomes without introducing foreign genetic materials, meeting the basic safety requirement for biomedical application of the engineered cells. Recently, *Wu et al.* have shown in mice that a dominant mutation in *Crygc* gene that causes cataracts could be rescued by Cas9 mediated DSB on the mutant allele which triggered homology-directed repair based on the endogenous WT allele, raising the hope for the application of current TALEN delivery technology to correct genetic diseases without the need for the introduction of exogenous DNA or RNA [23].

In this study, the ExoS54-TALEN fusion protein, about 100 KD in size, has been demonstrated to be injected through the T3SS of *P. aeruginosa*, further expanding the upper limit of the injectable exogenous proteins via the T3SS. The results further prove that the ExoS54 is a powerful signal peptide for the delivery of large exogenous proteins without interfering with their biological functions. Based on published studies, the efficiency of TALEN mediated target gene inactivation varies greatly and presumably influenced by a number of factors. First, the DNA binding domain of the TALEN, *i.e.* the targeting sequence and length, determines the efficiency of binding and processing of the target sequence. Second, the efficiency of plasmid transfection, which in our case reached about 60% as assessed by RFP expressing plasmid transfection control. Third, efficiency of the TALEN gene expression inside the host cell. Finally, stability of the TALEN protein inside the host cells. Transfection of the TALEN plasmid DNA resulted in approximately 20% of the HeLa-Venus cells lost fluorescence, while single bacterial injection of the TALEN protein pairs resulted in approximately 10% Venus negative cells. A plausible explanation for the difference is due to a shorter half-life of the bacterially injected TALEN proteins which survive inside the target cells for less than 12 hours (Fig. 2B). In contrary, the duration of TALEN protein expressed from plasmid DNA is much longer as the plasmid DNA can exist for at least several days within the transfected host cells [24]. Despite the low efficiency, short half lives of the bacterially injected TALEN proteins should in theory result in less off target cleavage, although plasmid transfection mediated delivery of TALEN has been shown to have very low off target cleavage [25].

Based on the cell synchronization studies, the TALEN protein pair tends to inactivate *venus* gene more efficiently in S phase cells, as delivery of the TALEN into S phase cells resulted in higher rate of the *venus* negative cells, consistent with our previous studies of Cre and MyoD deliveries [18,18]. Presumably, the target site of *venus* gene is more easily accessible to the TALENs during S phase. For non-S phase cells, as the intracellular half lives of the TALENs (<12 hrs) are less than half of a cell cycle, many of the intracellular TALENs may never had a chance to pass through the S phase. Following cell synchronization, S-phase HeLa-Venus cells reached more than 50% in cell population, more than two folds of S-phase cells in non-synchronized cells (20%). Accordingly, Venus negative cells also increased about two folds in synchronized cells (20%) compared to the non-synchronized cells (10%). This is consistent with the prediction that S-phase cells are much more susceptible to the injected TALEN proteins. Whether the injected TALENs target *venus* genes exclusively during S phase or simply S phase cells are more susceptible need to be clarified with additional experimental studies.

The efficiency of *venus* targeting can be increased further with repeated TALEN protein delivery. Indeed, with the second round of infection carried out at either MOI 50 or 100, a clear dose-dependent increases of non-fluorescent cell population were observed (Fig. 5A), suggesting that with multiple rounds of the protein delivery, any desired rates of target modifications should be achievable. Current limit is the residual cytotoxicity of the TALEN delivery strain, as excess rounds of injection causes significant cytotoxicity, thus obtaining a completely non-cytotoxic TALEN delivery strain is highly desirable.

This study provides a novel tool to deliver TALEN proteins into mammalian cells for the purpose of genome editing, without the need for delivery of genetic materials, meeting the basic safety requirements for biomedical application of the engineered cells. Additionally, efficiency of genome editing can be controlled by various infection parameters, such as MOI, duration of infection, number of repeated infections and cell cycle manipulation. The use of bacterial type III secretion system to deliver protein has the potential to replace many current methods of genome editing as well as directed cellular reprogramming. More recently, RNA and Cas9 mediated genome editing proved to be highly efficient, it will be interesting to test if bacterial protein secretion systems can be adapted for delivery of such RNA-protein complexes into mammalian cells.

## Materials and Methods

### Bacterial Strains and Plasmids

Bacterial strains and plasmid constructs used in this study are listed in Table 1. *P. aeruginosa* PAK-J deleted of *exoS*, *exoT* and *exoY* and PAK-J deleted of *popD* or *pscF* have previously been described [18]. The *P. aeruginosa* strains were grown in Luria broth (LB) or LB agar plates at 37°C. Antibiotics were used at a final concentration of 150Μg per ml carbenicillin for plasmid selection.

**Table 1:** Bacterial strains and plasmids.

*Strains or plasmids*	*Description*	*Ref.*
PAK-JΔ*STY*	PAK-J deleted of *exoS*, *exoT* and *exoY*	[18]
PAK-JΔ*popD*	PAK-J deleted by *popD*	[18]
PAK-JΔ*pscF*	PAK-J deleted by *pscF*	This study
pExoS54-Flag	pUCP19 with *exoS* promoter and N-terminal 54AA followed by a FLAG tag	[18]
pTAL3	TALEN cloning vector	[8]
pTAL-TALEN1	Plasmid encoding TALEN1 that targets left arm of *venus* gene	This study
pTAL-TALEN2	Plasmid encoding TALEN2 that targets right arm of *venus* gene	This study
pUCP19	Cloning vector for *P. aeruginosa*	[26]
pExoS54-Flag-TALEN1	pUCP19 with Exos54-Flag-TALEN1 fusion coding sequence	This study
pExoS54-Flag-TALEN2	pUCP19 with Exos54-Flag-TALEN2 fusion coding sequence	This study
pBCAG-Venus	Plasmid encoding *venus* gene	This study
pBCAG-RFP	Plasmid encoding *rfp* gene	This study

The *venus* gene targeting TALEN pair were constructed in pTAL3 vector following the detailed instruction provided by Golden Gate cloning kit from Voytas laboratory [8 and AddGene]. The *venus* gene binding targeting sequences are shown in Fig. 1A. The ExoS54-Flag-TALEN fusion constructs were generated by in frame fusion of the whole TALEN coding sequence to the pExoS54-Flag which has previously been described [18]. A Venus expressing plasmid, pBCAG-Venus, was used to transfect HeLa cell and selected for puromycin resistance. Stable Venus positive cells were subjected to single cell cloning following FACS sort and a single copy Venus gene integrant was designated as HeLa-Venus cell line. The TALEN targeting sequence within Venus gene was amplified using PCR primers (Forward: 5′-ATGGTGAGCAAGGGCGAGGAG-3′; Reverse: 5′-GCCCTCGAACTTCACCTCGGCG-3′), cloned into pGEM-T Easy (Promega) vector and subjected to sequence analysis.

### Cell Culture

Human cervix epithelial cell line HeLa (ATCC CCL-2) was grown in Dulbecco’s Modified Eagle Medium (DMEM; Gibco) supplemented with 10% heat-inactivated Fetal Bovine Serum (FBS). Cells were cultured at 37°C with 5% CO_2_ and supplemented with penicillin and streptomycin (Cellgro). HeLa cells were transfected with pBCAG-Venus and selected for Puromycin resistance (2 µg/ml), obtaining stable HeLa-Venus cells through single cell cloning. Cells were infected with bacteria in DMEM+10%FBS containing no antibodies. Ciprofloxacin was added at final concentration of 25 µg per ml for elimination of the TALEN delivery bacterial cells.

### Transfection of TALEN Plasmids

HeLa or HeLa-Venus cells were seeded in 12-well plates at 40–80% confluency one day before transfection. Plasmid DNA (0.6 µg), purified with Qiagen Plasmid Kit, was diluted with cell growth medium without serum or antibiotics to a final volume of 40 µl. Then, 4.8 µl of PolyFect Transfection Reagent (QIANGEN) was added to the DNA solution, mixed and incubated at room temperature for 5–10 min. The mixture was then added to the cell cultures slowly. Cells were incubated at 37°C and 5% CO_2_ for at least 16 hours before downstream experiments.

### Protein Injection Assay

HeLa cells were seeded at approximately 70% confluence in antibiotic-free medium. *P. aeruginosa* strains were grown at 37°C in Luria broth containing carbenicillin until reaching an optical density (OD_600_) of 0.8. HeLa cells were co-cultured with bacteria at a multiplicity of infection (MOI) of 100 for 3 hours. Infection was terminated by washing cells in PBS for three times and growing the cells on DMEM +10% FBS containing 25 µg per ml ciprofloxacin.

For Western Blot analysis of injected protein, cells were collected at indicated time points post bacterial infection by incubation in 0.25% trypsin for 5 min and centrifuged at 500×g for 10 min. The nuclear and cytoplasmic protein extracts were prepared using an extraction kit from Beyotime and followed the manufacturer’s instructions. All the protein samples were mixed with SDS-PAGE loading buffer and boiled for 10 min. Following separation on 10% SDS-PAGE and transfer onto polyvinylidene fluoride (PVDF) membrane, the blots were probed with antibody against Flag (mouse M2 monoclonal Ab, Sigma).

### Flow Cytometry

Cells were treated with 0.25% trypsin for 5 min and collected by centrifugation at 500×g for 10 min. Cells were fixed by 4% formaldehyde in PBS for 30 min at room temperature, washed once with PBS and resuspended in PBS. Cells were analyzed for Venus fluorescence using FACS Aria II (BD-Biosciences).

### Cell Synchronization

Cells were grown in DMEM containing 10% FBS for 2 days, followed by growth in medium containing 2 mM thymidine for 18 hours. After washing twice with PBS, the cells were incubated in regular medium for 9 hours and then cultured again in 2 mM thymidine for 17 hours. At 0, 4, 6, 8, 12, 18 and 24 hours after removing the second thymidine block, the cells were fixed by ice-cold 70% ethanol for 24 hours, then centrifuged and resuspended in 0.4 ml of PBS containing 50 µl RNase A (10 mg/ml) and 10 µl of PI (2 mg/ml) at 37°C for 30 min in dark. The cell-cycle phase distribution was analyzed by flow cytometer.
